# First‐In‐Human Study to Assess the Pharmacokinetics and Safety of DS‐6016a After Single Subcutaneous Injection in Healthy Japanese Adults

**DOI:** 10.1002/cpdd.70023

**Published:** 2026-01-21

**Authors:** Kei Okita, Hidetoshi Furuie, Akifumi Kurata, Yasuko Owada, Satoshi Yoshiba, Kei Furihata, Takaaki Oka, Yushi Kashihara, Hitoshi Ishizuka, Kazutaka Yoshihara

**Affiliations:** ^1^ Quantitative Clinical Pharmacology Department Daiichi Sankyo Co., Ltd Tokyo Japan; ^2^ Medical Corporation Heishinkai OPHAC Hospital Osaka Japan; ^3^ Data Intelligence Department Daiichi Sankyo Co., Ltd Tokyo Japan

**Keywords:** ALK2 inhibitor, DS‐6016a, fibrodysplasia ossificans progressiva, first‐in‐human, pharmacokinetics

## Abstract

Fibrodysplasia ossificans progressiva is a rare, progressive autosomal dominant genetic disease caused by an activin receptor‐like kinase 2 (ALK2) mutation with a need for effective prophylactic therapies. This single‐center, randomized, double‐blind, placebo‐controlled study evaluated the pharmacokinetics and safety of DS‐6016a, a novel humanized monoclonal anti‐ALK2 antibody, in healthy Japanese adults. In each of the 5–1000 mg DS‐6016a or placebo single subcutaneous dose cohorts, eight male participants were randomly assigned at a 3:1 ratio. DS‐6016a had median times to maximum plasma concentration of 144–240 h and elimination half‐lives of 391–844 h. The increase in DS‐6016a exposure was greater than dose‐proportional across the dose range of 5–1000 mg. The incidence of study drug‐related treatment‐emergent adverse events (TEAEs) was 17% in the placebo group and 11% across all DS‐6016a groups; all were mild and resolved without treatment. No deaths, serious or severe TEAEs, or TEAEs leading to study discontinuation were reported. Ferritin showed a statistically significant dose‐dependent decrease from baseline, while serum iron showed no clear dose‐dependency. No relationship was observed between DS‐6016a dose and the development of anti‐drug antibodies. DS‐6016a had an acceptable safety profile at single subcutaneous doses of 5–1000 mg.

Fibrodysplasia ossificans progressiva (FOP) is a progressive autosomal dominant genetic disease in which bone tissue forms ectopically in soft tissues such as skeletal muscles, tendons, and ligaments, a process that does not usually occur in healthy individuals.^1^ FOP is a rare disease that affects about 1 in 2 million people worldwide, regardless of race, geographical region, or sex.^1^, ^2^

FOP is caused by a mutation (617G>A; R206H) in activin receptor‐like kinase 2 (ALK2), also known as activin A receptor type 1, which is a bone morphogenetic protein (BMP) type 1 receptor.^2^, ^3^ While atypical cases with other ALK2 mutations have recently been reported, approximately 95% of patients with FOP have the mutation resulting in R206H.^3^ In addition to hyperactive BMP signaling induced by mutant ALK2, ligands that normally do not enhance BMP signals, such as activin A, have been reported to bind to mutant ALK2, leading to excessive signaling and the onset of heterotopic ossification in FOP. Removing this ossification is difficult as invasive medical procedures such as intramuscular injection, biopsy, and surgery are contraindicated in patients with FOP.^4^

Palovarotene was approved in Canada in 2022 and in the US in 2023 for the treatment of FOP and is indicated for the reduction in volume of new heterotopic ossification in adults and pediatric patients aged ≥8 years for females and ≥10 years for males with FOP.^5^ Palovarotene is an orally bioavailable retinoid that acts as a retinoic acid receptor (RAR) agonist with particular selectivity for the gamma subtype of RAR. However, it is extremely difficult to remove the formed ectopic bone tissue with drug therapy, so there is an unmet need for the development of agents that prophylactically inhibit ectopic ossification in FOP.

Rm0443, an anti‐ALK2 antibody, was found to prevent ectopic ossification in a mouse model of FOP with the human R206H variant.^6^ Therefore, anti‐ALK2 antibodies, such as the novel humanized monoclonal antibody DS‐6016a, may be clinically useful therapeutic agents for FOP, with possible utility for the prophylactic suppression of ectopic ossification.

Administration of an ALK2 inhibitor, such as KER‐047, has been shown to decrease ferritin and increase serum iron.^7^ The mechanism is thought to be that BMP signaling is strongly upregulated in response to increased hepatic iron levels, promoting the production of hepcidin to prevent further release of iron into circulation.^8^ In particular, BMP‐6 is an iron‐sensing molecule that stimulates the expression of hepcidin and induces the degradation of ferroportin, an iron‐regulating transporter expressed on iron‐utilizing cells and enterocytes. Furthermore, iron concentrations increase when blood hepcidin levels decrease. DS‐6016a binds to ALK2 and blocks this BMP signal pathway, which may decrease hepcidin, stabilize ferroportin, and increase blood iron concentrations. DS‐6016a is thought to bind to and inhibit ALK2 similarly to KER‐047, but it is unclear whether the binding site and the extent of inhibition are the same. In nonclinical studies of DS‐6016a, an increase in serum iron and a decrease in unsaturated iron‐binding capacity (UIBC) have been observed in relatively high dose levels. However, they have been considered toxicologically insignificant because of a lack of histopathological findings suggestive of organ injury attributable to iron excess.

This study aimed to evaluate the pharmacokinetics (PK) and safety of DS‐6016a after single subcutaneous administration in healthy Japanese adults, along with the formation of anti‐drug antibodies (ADAs) against DS‐6016a. Furthermore, iron‐related parameters (serum iron, UIBC, and ferritin) were also evaluated in a clinical setting.

## Methods

### Study Design

This single‐center, randomized, double‐blind (sponsor‐unblinded), placebo‐controlled, between‐group dose‐escalation study was conducted at OPHAC Hospital, Medical Corporation Heishinkai, in Osaka, Japan, from March 19, 2021 to June 30, 2023. Study participants were admitted to the hospital the day before the administration of a single dose of the study drug. The duration of hospitalization was 11 days (10 nights), and post‐study examinations were conducted 56 days after the treatment.

This study was pre‐approved by Medical Corporation Heishinkai OPHAC Hospital IRB (approval date, March 19, 2021) and was registered at ClinicalTrials.gov and Japan Registry of Clinical Trials under the identifier numbers NCT04818398 and jRCT2051200155, respectively. This study was also conducted in accordance with the ethical principles of the Declaration of Helsinki, and all study participants provided written informed consent. This clinical trial was conducted in compliance with the standards stipulated in Article 14, Paragraph 3, and Article 80‐2 of the Pharmaceuticals and Medical Devices Act, as well as the Ministerial Ordinance on Standards for Conducting Clinical Trials of Pharmaceuticals, Ministry of Health, Labor and Welfare Ordinance No. 28 dated March 27, 1997.

### Study Participants

Participants had to satisfy all of the following criteria to be included in the study: healthy Japanese male adult, aged 20–45 years at the time of informed consent, with a body mass index ≥18.5 to <25.0 kg/m^2^ at screening.

The main exclusion criteria were as follows: history of a serious disease (central nervous system, cardiovascular, respiratory, blood and hematopoietic, gastrointestinal system disorders, or other disorders); clinically significant symptoms (e.g., headache, dizziness), findings (e.g., blood pressure decreased), abnormalities on electrocardiogram (ECG), or abnormal laboratory values; history of hypersensitivity to any drugs; amino acid mutation(s) of ALK2; or any condition the investigator or subinvestigator otherwise considered inappropriate for the study.

### Randomization and Blinding

This was a double‐blind study in which all those who were involved in the study were blinded, with the following exceptions: the independent biostatistician, the independent person responsible for statistics, the drug concentration measurement laboratory staff members, the person responsible for the study drug assignment at the study site, and the study drug assignment staff at the study site. During the cohort transition evaluation, the Clinical Scientist, Medical Specialist, and Clinical Study Director were authorized to be unblinded only under specific conditions, such as if safety concerns were raised that required knowledge about treatment allocation. However, no safety concerns were raised in this study; thus, no such unblinding occurred.

The independent biostatistician prepared a randomization schedule that listed participant numbers and the corresponding study drug (DS‐6016a or placebo), then used the permuted block method for randomization to DS‐6016a or placebo with a ratio of 3:1. The independent biostatistician submitted the sealed randomization schedule for each cohort to the person responsible for study drug assignment at the study site before the start of study drug administration in each cohort.

### Intervention

This study consisted of six dose‐escalation cohorts. In each cohort, eight participants were randomly assigned to receive DS‐6016a or placebo at a 3:1 ratio (5‐, 15‐, 50‐, 150‐, 500‐, and 1000‐mg Cohorts).

Selection of doses was based on safety margins calculated from in vivo nonclinical studies in mice and cynomolgus monkeys (DaiichiSankyo Co. Ltd, unpublished data).

The study started with the 5‐mg cohort and proceeded to the next cohort. The first two participants in each cohort undergoing sentinel dosing were randomly assigned 1:1 to receive DS‐6016a or placebo. Adverse events (AEs) that occur immediately after dosing were evaluated in the first two participants, and it was confirmed that there were no safety concerns within the first 24 h after administration. After that, the remaining six participants (DS‐6016a:placebo = 5:1) received the study drug. Participants from each cohort were monitored for 14 days after drug administration; safety and/or PK data were reviewed by the investigator, sponsor, and medical expert before the next cohort was administered the next dose. The appropriateness of starting administration from Cohort 4 onward was determined based on the already available PK data. Up to Cohort 3, results of the PK evaluations were not required for proceeding to the next cohort, but when available, were used as reference information.

A single dose of the study drug (DS‐6016a or placebo) was subcutaneously injected across 1–5 sites on the abdomen. DS‐6016a was dissolved in water for injection. The study drug was administered at a concentration of 100 mg/mL, with a solution volume ranging from 0.05–10 mL. The use of any drugs (including supplements and vaccines) other than the study drug and any concomitant therapies was prohibited from 28 days before study drug administration to the end of the post‐study examination or follow‐up examination.

### Outcomes

The PK endpoints were C_max_, time to reach maximum plasma concentration (T_max_), AUC up to the last quantifiable time (AUC_last_), AUC up to time 336 h post‐dose (AUC_336 h_), apparent total body clearance (CL/F), AUC up to infinity (AUC_inf_), terminal elimination half‐life (t_1/2_), and apparent volume of distribution based on the terminal phase (V_z_/F) (CL/F, t_1/2_, and V_z_/F were to be calculated only if the elimination rate constants could be determined appropriately), as well as presence of ADAs.

The safety endpoints were: treatment‐emergent AEs (TEAEs), serious TEAEs, severe TEAEs, and TEAEs with a causal relationship with the study drug; laboratory tests, including iron‐related parameters (serum iron, UIBC, and ferritin); and body weight, vital signs (temperature, blood pressure, and pulse rate), and standard 12‐lead ECG.

### Pharmacokinetic and ADA Measurement Methods

Venous blood (4 mL) samples were collected from the antebrachial cutaneous vein at each time point into vacuum blood collection tubes containing ethylenediamine tetra‐acetic acid dipotassium salt. The samples were immediately inverted and cooled on ice. Plasma was obtained by centrifugation (at 4°C and 3000 rpm for 10 min), and approximately 1 mL aliquoted for testing and frozen, along with the remaining plasma. The specimens were cryopreserved (at ≤−20°C) until being sent to the laboratory (Shin Nippon Biomedical Laboratories, Ltd., Kagoshima, Japan) for measurement of concentrations of DS‐6016a, ADAs, and anti‐host cell protein antibodies by ligand binding assay.

To characterize the PK profile of DS‐6016a following subcutaneous dosing, plasma samples were collected at pre‐dose; at 2, 8, 24, and 36 h; and on days 2 (48 h), 3, 4, 5, 6, 7, 8, 9, 11, 14, 17, 21, 28, 35, 42, and 56 days (end of study) post‐dose or at early termination. The plasma concentration of DS‐6016a was measured by a validated ligand‐binding assay using the Gyrolab xP workstation (Gyros Protein Technologies AB, Uppsala, Sweden) at Shin Nippon Medical Laboratories, Ltd (Kagoshima, Japan). Additional details of the analytical method for plasma DS‐6016a are provided in the Supporting Information.

Two calibration ranges (i.e., the low and high ranges) were used for the analysis to fit a wide administered dose range. The calibration ranges for the low and high ranges were from 0.04 to 6 µg/mL and from 2.5 to 500 µg/mL, respectively. The intra‐run and inter‐run coefficients of variation around DS‐6016a nominal concentrations were less than 25% at the lower and upper limit of quantification and were less than 20% at the other concentrations in both the low and high ranges.

Immunogenicity evaluation was based on the assessment of ADAs in plasma collected at pre‐dose, on days 28 and 56, or at early termination. For subjects who were positive for ADAs at the time of the post‐study examination, an additional blood sample was collected for a follow‐up examination of ADAs every 3 months (±1 month). Additional blood samples were collected up to the earliest of the following time points: 1 year after the post‐study examination or confirmation of negative test results for ADA.

Plasma samples were analyzed for ADA binding to DS‐6016a using a validated electrochemiluminescence immunoassay at Shin Nippon Medical Laboratories, Ltd. The intra‐run and inter‐run coefficients of variation of this assay were lower than 20%, the drug tolerance level was 500 µg/mL, and the sensitivity was 4.69 ng/mL. ADAs were first detected, and then titers were assessed in the samples that were confirmed positive.

## Statistical Methods

The planned sample size was 48 participants (including six cohorts), based on the number of participants usually required for phase 1 studies. The PK analysis set included participants who received DS‐6016a as specified, had no major protocol deviations, and had plasma drug concentration measurements available. The safety analysis set included all participants who received a study drug (DS‐6016a or placebo) at least once. The ECG analysis set consisted of all participants who had received at least one dose of the study drug and had baseline and post‐dose ECG data.

Descriptive statistics were used to summarize participants’ demographic and clinical characteristics, including mean ± standard deviation (SD) or median (range) for continuous data and n (%) for categorical data. Summary statistics of plasma DS‐6016a concentrations were calculated for each dose and time point of blood collection, and a transition diagram of plasma DS‐6016a concentration was prepared. Additionally, summary statistics of PK parameters were calculated for each dose. The dose proportionality of DS‐6016a was evaluated using a power model to assess the relationship between the dose and PK parameters (C_max_ and AUC_last_). In addition, box plots were generated to assess the relationship between DS‐6016a dose levels and PK parameters.

For AEs, frequency tables were created by event and by causal relationship to the study drug. Frequency tables or shift tables were prepared for categorical data among laboratory test values, vital signs, body weight, and standard 12‐lead ECG, and summary statistics were calculated for quantitative data. AEs were grouped by system organ class and summarized by preferred term using Medical Dictionary for Regulatory Activities terminology (version 24.1).

For ferritin and serum iron, in addition to descriptive analyses, a post hoc analysis employing a mixed model for repeated measures (MMRM) was conducted to explore their mean response profiles over time and the dose‐response relationship. This model analyzed the change from baseline and included baseline value, dose (as a continuous variable), time (as a categorical variable), and the interaction between dose and time as fixed effects. To account for within‐subject correlations, an unstructured covariance matrix was applied. P‐values were derived from tests assessing the dose‐response relationship.

The incidence of ADAs was evaluated for each dose and antibody. The number and percentage of participants who developed or did not develop ADAs after the start of treatment were calculated. For each antibody, participants who were negative for ADAs at all time points after treatment with the study drug were defined as negative, and those who were positive for ADAs at one or more time points were defined as positive.

All statistical analyses were performed using SAS software version 9.4 (SAS Institute Inc., Cary, NC, USA). PK parameters were analyzed using WinNonlin software version 8.1 or higher (Certara, Inc., Radnor, PA, USA).

## Results

### Study Participants

Of the 107 participants who provided consent, 48 were randomized and received the study drug (two received a placebo and six received DS‐6016a in each of the six dose‐escalation cohorts). The main reasons for screening failure were failure to satisfy all inclusion criteria or fulfillment of exclusion criteria (39%, 23/59) and reaching maximum enrollment (32%, 19/59). All 48 randomized participants completed the study.

The baseline demographic and clinical characteristics of the study population are summarized in Table 1. No notable differences were found in the baseline demographic and clinical characteristics between the treatment groups. The baseline demographic and clinical characteristics of the PK analysis set were similar to those of the safety analysis set (data not shown).

**Table 1:** Baseline Demographic and Clinical Characteristics (Safety Analysis Set)

	Placebo	DS‐6016a dose (mg)						Total
	**n = 12**	**5** **n = 6**	**15** **n = 6**	**50** **n = 6**	**150** **n = 6**	**500** **n = 6**	**1000** **n = 6**	**N = 48**
Age, years	28 ± 5 (21–38)	33 ± 7 (22–41)	29 ± 5 (23–35)	27 ± 5 (20–33)	27 ± 9 (20–40)	27 ± 5 (21–36)	28 ± 5 (23–34)	28 ± 6 (20–41)
Weight, kg	64.4 ± 5.5	66.5 ± 6.9	62.2 ± 4.2	62.4 ± 9.0	59.4 ± 4.6	65.8 ± 6.6	64.9 ± 6.3	63.8 ± 6.2
Body mass index, kg/m^2^	22.0 ± 1.7	21.7 ± 1.6	20.9 ± 2.4	21.2 ± 1.9	20.0 ± 1.1	22.3 ± 1.9	22.1 ± 1.9	21.5 ± 1.8

Data are mean ± standard deviation and (range).

Placebo data are pooled from across the cohorts.

### Pharmacokinetics

**Figure 1:**
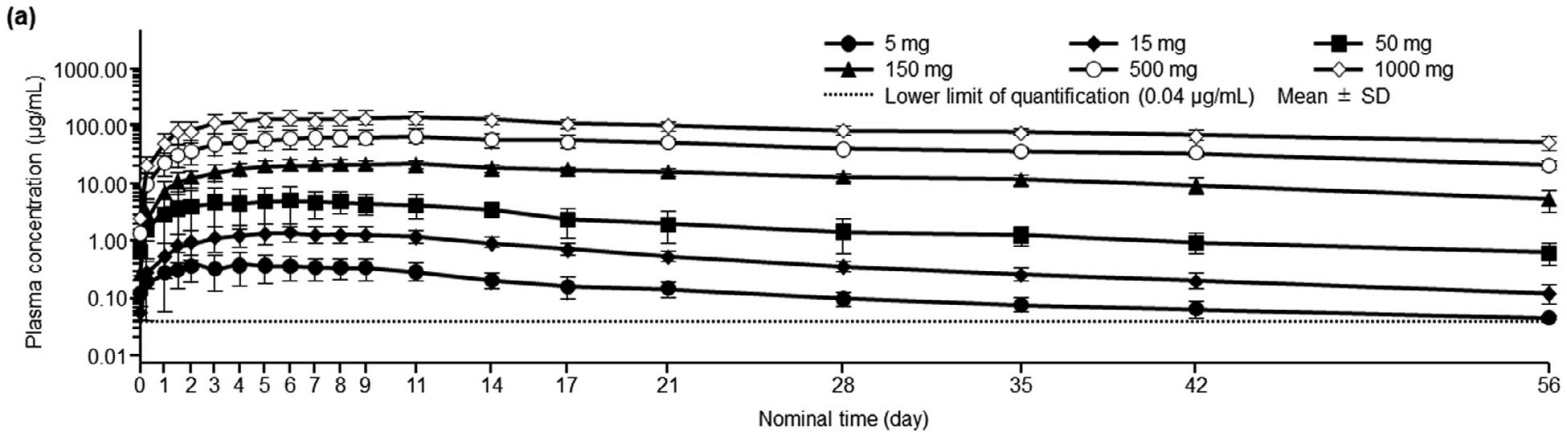
Time course change in plasma concentrations of DS‐6016a after single subcutaneous administration (pharmacokinetic analysis set): (a) semilogarithmic scale. SD, standard deviation.

The time course of the change in mean ± SD plasma concentrations of DS‐6016a after single subcutaneous administration is shown in Figure 1. DS‐6016a was slowly absorbed, with the median T_max_ ranging from 144 to 240 h; the plasma concentration of DS‐6016a slowly decreased after reaching C_max_, with the mean t_1/2_ ranging from 391 to 844 h, across a dose level range of 5–1000 mg. The mean C_max_ and AUC_last_ of DS‐6016a increased in a dose‐dependent manner, while the median T_max_ and mean t_1/2_ tended to increase across the DS‐6016a dose range of 5–1000 mg (Table 2).

**Table 2:** Pharmacokinetic Parameters of DS‐6016a (Pharmacokinetic Analysis Set)

	DS‐6016a dose (mg)											
	**5** **n = 6**		**15** **n = 6**		**50** **n = 6**		**150** **n = 6**		**500** **n = 6**		**1000** **n = 6**	
	**n**		**n**		**n**		**n**		**n**		**n**	
C_max_ (µg/mL)	6	0.44 ± 0.22	6	1.5 ± 0.5	6	5.8 ± 3.4	6	23.8 ± 3.5	6	74.3 ± 22.8	6	156 ± 39
AUC_last_ (µg·h/mL)	6	167 ± 87	6	738 ± 207	6	2790 ± 1380	6	17,700 ± 2580	6	58,900 ± 11,200	6	126,000 ± 24,200
AUC_336h_ (µg·h/mL)	6	105 ± 50	6	389 ± 131	6	1480 ± 780	6	6180 ± 1080	6	19,200 ± 6180	6	42,500 ± 11,500
AUC_inf_ (µg·h/mL)	3^a^, ^b^	263 ± 58	6	824 ± 245	6	3130 ± 1580	4^b^	19,400 ± 3490	1^b^	63,000 (NC)	0^b^	‐
T_max_ (h)	6	144 (36–264)	6	144 (120–264)	6	168 (72–216)	6	204 (144–264)	6	240 (168–264)	6	204 (144–408)
*t*_1/2_ (h)	4^a^ ^)^	413 ± 78	6	445 ± 86	6	391 ± 149	6	485 ± 175	6	643 ± 96	6	844 ± 258
CL/F (L/h)	3^a^, ^b^	0.0197 ± 0.0044	6	0.0197 ± 0.0062	6	0.0191 ± 0.0084	4^b^	0.00793 ± 0.00145	1^b^	0.00793 (NC)	0^b^	‐
V_z_/F (L)	3^a^, ^b^	10.7 ± 2.0	6	12.3 ± 3.3	6	9.7 ± 4.2	4^b^	4.4 ± 1.1	1^b^	5.8 (NC)	0^b^	‐

Data are mean ± standard deviation, except for T_max_, which is median (range).

^a^ Data for two participants were excluded because they did not satisfy the criteria for elimination rate constant calculation.

^b^ Data for participants whose percentage of extrapolated portion of AUC_inf_ were >20% were excluded.

AUC, area under the plasma concentration–time curve; AUC_inf_, area under the plasma concentration–time curve up to infinity; AUC_last,_ AUC up to the last quantifiable time; AUC_336h_, AUC up to time 336 h post‐dose; CL/F, apparent total body clearance; C_max_, maximum plasma concentration; NC, not calculated; QTcF, corrected QT by Fridericia's formula; T_max_, time to reach maximum plasma concentration; *t*_1/2_, terminal elimination half‐life; V_z_/F, apparent volume of distribution based on the terminal phase.

**Figure 2:**
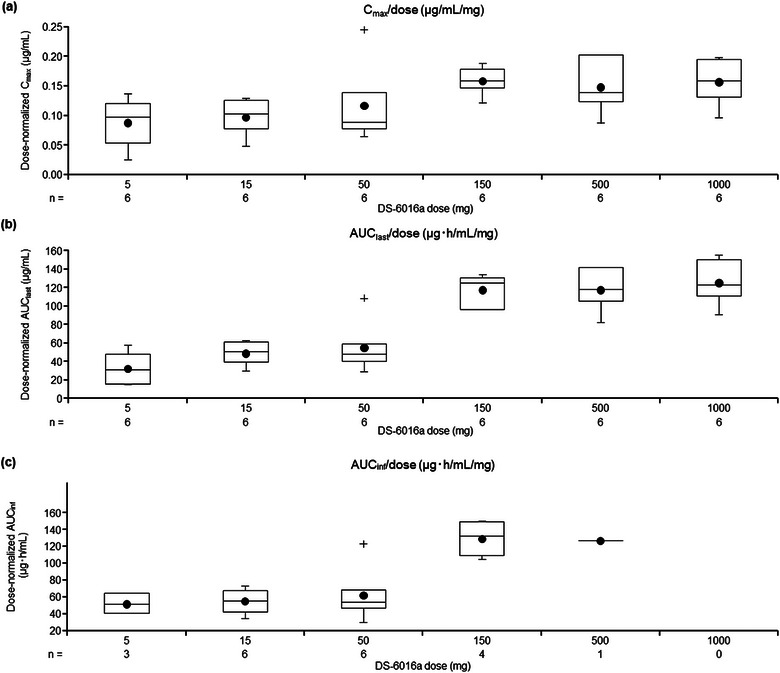
Relationship between the DS‐6016a dose and the pharmacokinetic parameters: (a) C_max_,(b) AUC_last_, and (c) AUC_inf_. The boxes represent the interquartile range (25^th^ to 75^th^ percentiles), the middle line indicates the median (50^th^ percentile), the black circle represents the mean, the top and bottom whiskers show the minimum and maximum values (excluding outliers), and the crosses represent outliers. AUCinf, area under the plasma concentration–time curve up to infinity; AUC_last_, area under the curve up to the last quantifiable time; C_max_, maximum plasma concentration.

The dose proportionality of DS‐6016a was assessed for C_max_, AUC_last_, and AUC_inf_ using a power model (Table 3). The increase in exposure was greater than dose‐proportional across the dose range of 5–1000 mg, and also tended to be linear at doses ≥150 mg for C_max_, AUC_last_, and AUC_inf_ (Figure 2a–c, respectively).

**Table 3:** Parameter Estimates of the Power Model for Assessing the Dose Proportionality of DS‐6016a (Pharmacokinetic Analysis Set)

		90% Confidence interval	
	Estimate	Lower	Upper
C_max_ (µg/mL)			
Intercept	−2.746	−3.031	−2.462
Slope	1.136	1.076	1.195
AUC_last_ (µg·h/mL)			
Intercept	3.012	2.748	3.277
Slope	1.282	1.227	1.337
AUC_inf_ (µg·h/mL)^a^			
Intercept	3.341	2.902	3.779
Slope	1.245	1.128	1.361

^a^ Data for participants whose percentages of extrapolated portion of AUC_inf_ were >20% were excluded.

AUC_inf_, area under the plasma concentration–time curve up to infinity; AUC_last,_ area under the plasma concentration–time curve up to the last quantifiable time; C_max_, maximum plasma concentration.

### Safety

The numbers and percentages of participants with TEAEs are summarized in Table S1. The incidence of TEAEs was 67% (8/12) in participants receiving placebo and 44% (16/36) in participants receiving DS‐6016a. The incidence of TEAEs related to the study drug was 17% (2/12) in the placebo group and 11% (4/36) in the DS‐6016a group. TEAEs related to the study drug were headache (17% [1/6] in the 15‐mg cohort), nausea (17% [1/6] in the 15‐mg cohort), rash (17% [1/6] in the 150‐mg cohort), alanine aminotransferase increased (8% [1/12] in the placebo group and 33% [2/6] in the 500‐mg cohort), aspartate aminotransferase increased (8% [1/12] in the placebo group), and gamma‐glutamyltransferase increased (8% [1/12] in the placebo group). Headache and nausea as TEAEs related to the study drug occurred in the same participant. All TEAEs related to the study drug were mild and resolved without treatment. No deaths, serious TEAEs, TEAEs leading to study discontinuation, or severe TEAEs were reported in this study.

**Figure 3:**
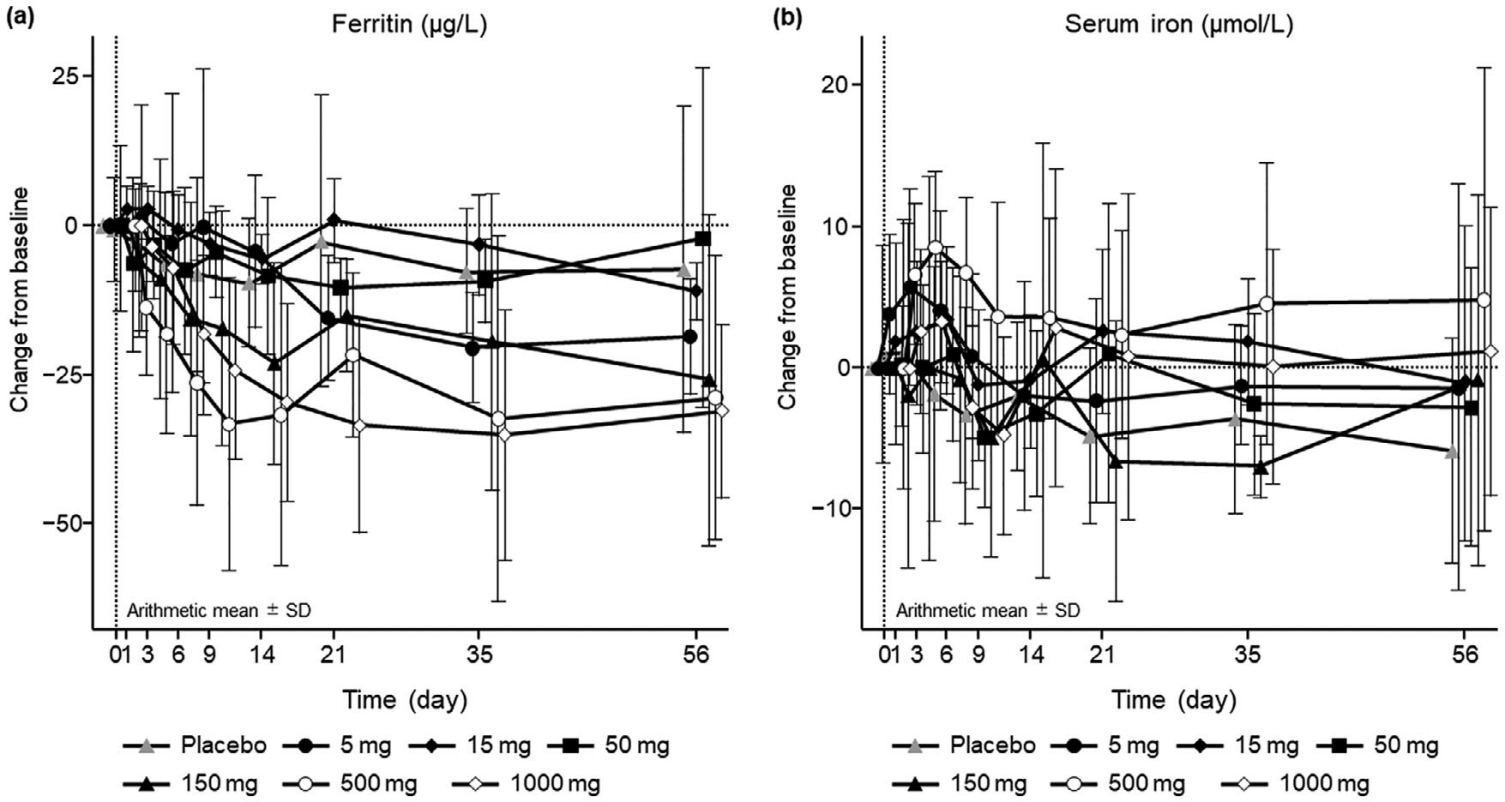
Change from baseline in (a) ferritin and (b) serum iron. SD, standard deviation.

No notable differences or trends were found among the treatment groups in the changes in the mean values over time in the serum chemistry test, including iron‐related serum chemistry parameters such as serum iron and UIBC. For ferritin, descriptive analysis of the mean profiles over time indicated a dose‐dependent decrease (Figure 3). This trend was supported by post hoc MMRM analysis, with *p*‐values below 0.05 at multiple post‐baseline visits. No clear dose‐dependent change was observed for serum iron (Figure 3; Table S2, Table S3, Figure S1, and Figure S2).

No notable trends or differences among the treatment groups were found in any of the laboratory parameters, vital signs, 12‐lead ECG, body weight, or other safety endpoints (data not shown).

### ADAs

No participants were positive for ADAs at baseline. After administration of the study drug, treatment‐induced ADA was reported in 11% of participants (4/36) who received DS‐6016a. The incidences of treatment‐induced ADA were 33% (2/6), 17% (1/6), 17% (1/6), 0% (0/6), 0% (0/6), and 0% (0/6) in participants receiving DS‐6016a in the 5‐, 15‐, 50‐, 150‐, 500‐, and 1000‐mg cohorts, respectively, and 0% (0/12) in the participants receiving placebo across all cohorts.

The individual AUC_last_ values of the four participants with ADA‐positive results were 75.9 µg·h/mL (5‐mg cohort), 137 µg·h/mL (5‐mg cohort), 618 µg·h/mL (15‐mg cohort), and 1480 µg·h/mL (50‐mg cohort). All of these values were lower than the mean AUC_last_ of the participants with ADA‐negative results in each dose cohort (i.e., 197, 763, and 3050 µg·h/mL in the 5‐, 15‐, and 50‐mg cohorts, respectively).

## Discussion

This phase 1 first‐in‐human study of DS‐6016a evaluated the PK and safety of DS‐6016a in healthy adults. Based on the results, Exposure to DS‐6016a exhibited a dose‐dependent increase in plasma exposure when administered subcutaneously at single doses of 5–1000 mg, with no critical safety concerns, particularly regarding iron‐related parameters.

Target‐mediated clearance is the process by which monoclonal antibodies are eliminated due to their specific binding to the target agent.^9^ After binding to their target agent, monoclonal antibodies may be internalized and catabolized through lysosomal degradation. Once the saturation dose level is reached or exceeded, target‐mediated clearance becomes negligible, and its elimination occurs through the nonspecific neonatal Fc receptor pathway. At low antibody drug concentrations relative to the antigen, clearance follows a linear pattern, while at higher antibody drug concentrations, clearance is non‐linear. With a further increase in antibody drug concentrations, linear clearance is observed once again.^9^ Based on the PK results of the present study, target‐mediated drug disposition due to ALK2 internalization is suggested, with ALK2 inhibition by DS‐6016a becoming saturated at doses above 150 mg. In addition, ferritin levels also exhibited a statistically significant dose‐dependent decrease across the dose range tested, with a generally saturated trend at doses ≥ 150 mg. These findings suggest that decreased ferritin levels may be a marker of ALK2 inhibition.

Ferritin is a key indicator of tissue iron stores, and low ferritin levels are commonly used to diagnose iron deficiency in clinical practice. In this study, the decrease in ferritin observed in normal participants is consistent with the administration of the ALK2 inhibitor KER‐047, which has been shown to decrease ferritin and increase serum iron.^7^ It is known that hepcidin binds to ferroportin and inhibits iron release,^10^ therefore based on the KER‐047 study,^7^ anti‐ALK2 antibodies may suppress ALK2‐BMP6 signaling, reduce hepcidin production, and promote ferroportin‐induced iron release from iron storage cells, leading to a decrease in ferritin levels.^8^, ^11^

DS‐6016a has raised concerns regarding potential toxicity due to increased serum iron and hemosiderin deposition in tissues, as observed in nonclinical studies, although these findings were considered to be toxicologically insignificant. While ferritin was decreased in this study, serum iron concentrations did not change significantly, so it is likely that iron is being removed from stores and uptake into reticulocyte hemoglobin (RET‐Hgb) is increased (Figure S3).^7^ Although no significant increase in serum iron was observed in the present study, an increasing trend was observed in the DS‐6016a group compared with the placebo group. One possible explanation for the lack of a significant increase in serum iron in this study is the activation of a feedback mechanism that regulates and maintains iron concentration. Iron in the body is distributed among the following: hemoglobin, ferritin, hemosiderin, myoglobin, tissue enzymes (heme and non‐heme iron), and transport iron.^12^ Under conditions of hepcidin suppression, iron may be used for erythropoiesis (incorporation into hemoglobin). KTI‐016 and KTI‐018, ALK2 neutralizing antibodies, have been shown to increase RET‐Hgb, decrease serum hepcidin, and increase serum iron in cynomolgus monkeys.^13^ The increase in RET‐Hgb was followed by increases in red blood cell hemoglobin and mean corpuscular hemoglobin concentration, suggesting that the mobilized serum iron is incorporated into the hemoglobin of the reticulocytes. The present study did not confirm the changes in hepcidin levels, RET‐Hgb, or hemosiderin deposition observed in nonclinical studies, and further investigation is needed to determine whether serum iron mobilized by the administration of DS‐6016a was used for erythropoiesis.

The present study has some limitations. First, the following were not evaluated: concentrations of iron‐containing proteins other than ferritin, including hepcidin and ferroportin; iron release from storage cells into the blood; RET‐Hgb content; and ALK2 receptor occupancy. Second, this study only included healthy male adults, and further investigation is warranted in female participants. Third, because this study involved single‐dose administration, changes associated with long‐term use were not evaluated. Finally, the PK sampling schedule was insufficient. Full drug elimination was expected by day 56 post‐dose, based on nonclinical data. However, the clinical data revealed a higher exposure and longer half‐life than that predicted from the nonclinical data. Based on these findings, the possibility of adjusting the sampling schedule has been considered for future clinical studies.

## Conclusions

Exposure to DS‐6016a increased in a dose‐dependent manner when administered subcutaneously at single doses of 5, 15, 50, 150, 500, and 1000 mg in healthy Japanese male adults. Overall, it had an acceptable safety profile, and no relationship was observed between DS‐6016a dose and the development of ADAs. Further studies are warranted to evaluate the efficacy and safety of DS‐6016a for the treatment of FOP.

## Funding

This study was funded by Daiichi Sankyo Co., Ltd.

## Conflicts of Interest

Kei Okita, Akifumi Kurata, Satoshi Yoshiba, Kei Furihata, Takaaki Oka, Yushi Kashihara, Hitoshi Ishizuka, and Kazutaka Yoshihara are employees of Daiichi Sankyo Co., Ltd. Hidetoshi Furuie and Yasuko Owada have no conflicts of interest to disclose.

## Supporting information



Supporting Information

## Data Availability

All de‐identified patient data relevant to this study are included in this article. Additional data and supporting documents pertaining to this study may be provided upon reasonable request made via this web address (https://vivli.org/ourmember/daiichi‐sankyo/) in accordance with the data sharing policy of Daiichi Sankyo Co., Ltd.
