# Supplementary material for: First‐In‐Human Study to Assess the Pharmacokinetics and Safety of DS‐6016a After Single Subcutaneous Injection in Healthy Japanese Adults
Source: Clin Pharmacol Drug Dev. 2026 Jan 21;15(1):e70023. doi: 10.1002/cpdd.70023 (PMC12821564; doi:10.1002/cpdd.70023)
Supplement: Supplementary file 1 — Supporting Information [file CPDD-15-0-s001.docx]

**Supplemental Information**

**Supplemental Methods.** Method for plasma DS-6016a analysis.

A bioanalytical method for the analysis of DS-6016a in human plasma containing dipotassium ethylenediaminetetraacetic acid using the Gyrolab xP workstation (Gyros Protein Technologies AB, Uppsala, Sweden) was developed and validated.

***Method Description***

Intact DS-6016a was quantitatively measured from human plasma using Gyrolab. In this assay, the primary capture reagent, biotinylated human activin receptor-like kinase 2 (ALK2)-His, binds to streptavidin-coated beads in the affinity column of a Bioaffy compact disc (CD). When plasma samples are injected into the column, intact DS-6016a present in samples binds to biotinylated human ALK2-His. Detection is achieved using Dylight labeled rabbit anti-DS-6016a antibody which binds to captured intact DS-6016a. Wash steps are performed between each reagent/sample addition to remove any unbound material. The CD is read by a laser that excites the DyLight, producing a fluorescent signal that is directly proportional to the concentration of intact DS-6016a.

***Reagents and Chemicals***

- Biotinylated human ALK2-His
- Dylight rabbit anti-DS-6016a antibody
- Tween-20 Surfact-Amps 20, 10% Solution, Thermo Fisher Scientific Inc. (Massachusetts, USA)
- Rexxip F, Gyros Protein Technologies (Uppsala, Sweden)
- Assay Diluent, ANP Technologies Inc. (Delaware, USA)
- MultiScreenHTS BV Filter Plate, 1.2 µm, clear, sterile, Millipore (Massachusetts, USA)
- Gyrolab Wash Buffer pH 11, Gyros Protein Technologies (Uppsala, Sweden)

***Equipment and Materials***

- Gyrolab xP workstation, Gyros Protein Technologies (Uppsala, Sweden)
- Gyrolab Control (version 7.2.2, Gyros), Gyrolab Evaluator (version 3.4.0, Gyros), and Watson-LIMS (version 7.4.1, Thermo Fisher Scientific Inc.) were used as analysis and control software.
- Gyrolab Bioaffy 1000 CD, Gyros Protein Technologies (Uppsala, Sweden)
- 0.200 mL skirted 96-well polymerase chain reaction (PCR) plates, Thermo Scientific Inc. (Massachusetts, USA)
- Microplate foil plate sealers, Gyros Protein Technologies (Uppsala, Sweden)

***Procedure***

1. Mix each centrifuged sample with assay buffer and load samples to 96-well filter plates.
2. Transfer each sample into the appropriate wells of the MultiScreenHTS BV filter plate. Centrifuge the filter plate/PCR plate assembly at 2000 rpm for 2 minutes at room temperature. Remove and discard the MultiScreenHTS BV filter plate, and seal the PCR plate.
3. Prepare biotinylated human ALK2-His working solution (capture reagent).
4. Prepare DyLight labeled rabbit anti-DS-6016a antibody working solution (detection reagent).
5. Add the working solutions and wash solution (0.1% Tween 20/1 × PBS) to the reagent plate.
6. Load the sample plates and reagent plates onto the Gyrolab xP workstation.

***Calculation of Concentrations***

Analyte concentrations were determined by applying mean response in duplicate analysis to the calibration curve. The concentration was multiplied by the dilution factor and then used as the measured concentration.

***Limits of Quantitation and Calibration Ranges***

The lower limit of quantitation was the lowest non-zero concentration level that was quantified with acceptable accuracy and precision. The upper limit of quantitation was the highest non-zero concentration level that was quantified with acceptable accuracy and precision. Two calibration ranges (i.e., the low and high ranges) were used for the analysis to fit for a wide administered dose range. The calibration ranges for the low and high ranges were from 0.04 µg/mL to 6 µg/mL, and from 2.5 µg/mL to 500 µg/mL, respectively.

***Accuracy and Precision***

Accuracy and precision were evaluated by analyzing quality control pools prepared at 0.04, 0.12, 1.2, 4.8, and 6 µg/mL for low range, and 2.5, 7.5, 60, 300 and 500 µg/mL for high range by fortifying blank matrix pools with the reference standard. Precision was expressed as the percent coefficient of variation for each pool. Accuracy was measured as the percent difference from the theoretical value. The intra-run and inter-run coefficients of variation around DS-6016a nominal concentrations were <25% at the lower and upper limit of quantifications and were <20% at the other concentrations in both the low and high ranges. The intra-run and inter-run coefficients of variation were 1.2% to 4.2% and 5.0% to 8.3% in the low range method. Those values in the high range method were 1.2% to 4.7% and 3.4% to 12.1%, respectively.

**Supplemental Table S1.** Summary of TEAEs (Safety Analysis Set)

|  | **Placebo** | **DS-6016a dose (mg)** | | | | | | | **Total** |
| --- | --- | --- | --- | --- | --- | --- | --- | --- | --- |
|  | **n = 12** | **5**  **n = 6** | **15**  **n = 6** | **50**  **n = 6** | **150**  **n = 6** | **500**  **n = 6** | **1000**  **n = 6** | **All**  **n = 36** | **N = 48** |
| TEAEs | 8 (66.7) | 2 (33.3) | 3 (50.0) | 3 (50.0) | 2 (33.3) | 4 (66.7) | 2 (33.3) | 16 (44.4) | 24 (50.0) |
| TEAEs related to the study drug | 2 (16.7) | 0 (0.0) | 1 (16.7) | 0 (0.0) | 1 (16.7) | 2 (33.3) | 0 (0.0) | 4 (11.1) | 6 (12.5) |
| Severe TEAEs | 0 (0.0) | 0 (0.0) | 0 (0.0) | 0 (0.0) | 0 (0.0) | 0 (0.0) | 0 (0.0) | 0 (0.0) | 0 (0.0) |
| Serious TEAEs | 0 (0.0) | 0 (0.0) | 0 (0.0) | 0 (0.0) | 0 (0.0) | 0 (0.0) | 0 (0.0) | 0 (0.0) | 0 (0.0) |
| TEAEs leading to discontinuation | 0 (0.0) | 0 (0.0) | 0 (0.0) | 0 (0.0) | 0 (0.0) | 0 (0.0) | 0 (0.0) | 0 (0.0) | 0 (0.0) |
| Death (TEAEs leading to fatal outcome) | 0 (0.0) | 0 (0.0) | 0 (0.0) | 0 (0.0) | 0 (0.0) | 0 (0.0) | 0 (0.0) | 0 (0.0) | 0 (0.0) |

Data are n (%).

TEAEs, treatment-emergent adverse events.

**Supplemental Table S2.** Time course of change from baseline in Ferritin (Safety Analysis Set)

|  | | Treatment group | | | | | | |  |
| --- | --- | --- | --- | --- | --- | --- | --- | --- | --- |
|  | | Placebo (N=12) | 5 mg (N=6) | 15 mg (N=6) | 50 mg (N=6) | 150 mg (N=6) | 500 mg (N=6) | 1000 mg (N=6) | p-value |
| Day 2 | Model-based mean | -3.82 | -3.81 | -3.79 | -3.74 | -3.58 | -3.03 | -2.25 | 0.7107 |
|  | 95% CI | (-7.11, -0.53) | (-7.08, -0.55) | (-7.01, -0.57) | (-6.81, -0.67) | (-6.38, -0.79) | (-6.68, 0.62) | (-9.43, 4.94) |  |
| Day 4 | Model-based mean | -4.07 | -4.08 | -4.11 | -4.21 | -4.49 | -5.48 | -6.89 | 0.6010 |
|  | 95% CI | (-8.31, 0.17) | (-8.29, 0.13) | (-8.26, 0.04) | (-8.18, -0.24) | (-8.11, -0.87) | (-10.17, -0.79) | (-16.07, 2.29) |  |
| Day 7 | Model-based mean | -8.33 | -8.38 | -8.48 | -8.83 | -9.83 | -13.35 | -18.38 | 0.1086 |
|  | 95% CI | (-13.20, -3.45) | (-13.22, -3.53) | (-13.26, -3.70) | (-13.40, -4.26) | (-14.00, -5.67) | (-18.75, -7.96) | (-28.91, -7.85) |  |
| Day 10 | Model-based mean | -8.52 | -8.60 | -8.78 | -9.38 | -11.11 | -17.15 | -25.78 | 0.0185 |
|  | 95% CI | (-14.15, -2.88) | (-14.20, -3.01) | (-14.30, -3.26) | (-14.66, -4.10) | (-15.93, -6.29) | (-23.38, -10.92) | (-37.91, -13.64) |  |
| Day 15 | Model-based mean | -11.30 | -11.39 | -11.57 | -12.20 | -14.00 | -20.30 | -29.31 | 0.0034 |
|  | 95% CI | (-15.93, -6.66) | (-15.99, -6.78) | (-16.11, -7.02) | (-16.54, -7.85) | (-17.96, -10.04) | (-25.44, -15.17) | (-39.33, -19.29) |  |
| Day 22 | Model-based mean | -8.08 | -8.18 | -8.39 | -9.11 | -11.17 | -18.37 | -28.67 | 0.0031 |
|  | 95% CI | (-13.33, -2.83) | (-13.40, -2.97) | (-13.54, -3.24) | (-14.03, -4.19) | (-15.66, -6.68) | (-24.19, -12.56) | (-40.00, -17.33) |  |
| Day 36 | Model-based mean | -12.17 | -12.28 | -12.49 | -13.22 | -15.31 | -22.64 | -33.10 | 0.0021 |
|  | 95% CI | (-17.29, -7.06) | (-17.36, -7.20) | (-17.50, -7.47) | (-18.02, -8.42) | (-19.69, -10.94) | (-28.28, -16.99) | (-44.10, -22.11) |  |
| Day 57 | Model-based mean | -13.03 | -13.11 | -13.27 | -13.83 | -15.42 | -20.99 | -28.96 | 0.0695 |
|  | 95% CI | (-19.89, -6.17) | (-19.92, -6.30) | (-19.99, -6.55) | (-20.25, -7.40) | (-21.28, -9.56) | (-28.53, -13.46) | (-43.63, -14.28) |  |
| Model-based means and associated 95% Cs are derived from a mixed model for repeated measures for change from baseline. The model includes baseline value (continuous), dose (continuous), time (categorical), and the interaction between dose and time as fixed effects. An unstructured covariance structure is assumed for the within-subject covariance. P-value is based on a t-test of the parameter representing the effect of dose on the change from baseline, specifically assessing the dose-response relationship. | | | | | | | | | |

**Supplemental Table S3.** Time course of change from baseline in serum iron (Safety Analysis Set)

|  | | Treatment group | | | | | | |  |
| --- | --- | --- | --- | --- | --- | --- | --- | --- | --- |
|  | | Placebo (N=12) | 5 mg (N=6) | 15 mg (N=6) | 50 mg (N=6) | 150 mg (N=6) | 500 mg (N=6) | 1000 mg (N=6) | p-value |
| Day 2 | Model-based mean | 1.08 | 1.10 | 1.13 | 1.26 | 1.61 | 2.86 | 4.64 | 0.1138 |
|  | 95% CI | (-0.69, 2.86) | (-0.67, 2.86) | (-0.61, 2.87) | (-0.41, 2.92) | (0.09, 3.14) | (0.90, 4.82) | (0.84, 8.43) |  |
| Day 4 | Model-based mean | 1.85 | 1.87 | 1.91 | 2.05 | 2.46 | 3.87 | 5.89 | 0.1404 |
|  | 95% CI | (-0.31, 4.02) | (-0.28, 4.02) | (-0.21, 4.03) | (0.02, 4.09) | (0.60, 4.32) | (1.48, 6.26) | (1.26, 10.51) |  |
| Day 7 | Model-based mean | 1.06 | 1.06 | 1.06 | 1.04 | 1.01 | 0.87 | 0.67 | 0.8684 |
|  | 95% CI | (-0.83, 2.96) | (-0.82, 2.95) | (-0.80, 2.92) | (-0.74, 2.83) | (-0.62, 2.64) | (-1.23, 2.96) | (-3.39, 4.73) |  |
| Day 10 | Model-based mean | -2.43 | -2.43 | -2.42 | -2.39 | -2.31 | -2.04 | -1.65 | 0.7074 |
|  | 95% CI | (-4.10, -0.76) | (-4.08, -0.77) | (-4.05, -0.79) | (-3.95, -0.83) | (-3.74, -0.88) | (-3.88, -0.20) | (-5.21, 1.92) |  |
| Day 15 | Model-based mean | -1.85 | -1.82 | -1.75 | -1.52 | -0.86 | 1.44 | 4.74 | 0.0372 |
|  | 95% CI | (-4.31, 0.61) | (-4.26, 0.62) | (-4.16, 0.66) | (-3.83, 0.79) | (-2.97, 1.25) | (-1.28, 4.16) | (-0.54, 10.01) |  |
| Day 22 | Model-based mean | -2.54 | -2.52 | -2.47 | -2.30 | -1.81 | -0.10 | 2.34 | 0.0947 |
|  | 95% CI | (-4.84, -0.24) | (-4.80, -0.23) | (-4.73, -0.21) | (-4.46, -0.14) | (-3.78, 0.16) | (-2.64, 2.44) | (-2.58, 7.27) |  |
| Day 36 | Model-based mean | -2.18 | -2.15 | -2.10 | -1.94 | -1.46 | 0.21 | 2.59 | 0.0943 |
|  | 95% CI | (-4.44, 0.09) | (-4.40, 0.10) | (-4.33, 0.12) | (-4.07, 0.19) | (-3.41, 0.49) | (-2.28, 2.70) | (-2.21, 7.39) |  |
| Day 57 | Model-based mean | -2.88 | -2.85 | -2.78 | -2.53 | -1.81 | 0.68 | 4.25 | 0.0949 |
|  | 95% CI | (-6.25, 0.49) | (-6.20, 0.50) | (-6.08, 0.53) | (-5.69, 0.63) | (-4.70, 1.07) | (-3.02, 4.39) | (-2.93, 11.43) |  |
| Model-based means and associated 95% CIs are derived from a mixed model for repeated measures for change from baseline. The model includes baseline value (continuous), dose (continuous), time (categorical), and the interaction between dose and time as fixed effects. An unstructured covariance structure is assumed for the within-subject covariance. P-value is based on a t-test of the parameter representing the effect of dose on the change from baseline, specifically assessing the dose-response relationship. | | | | | | | | | |

**Supplemental Figure S1.** Time course of change from baseline in Ferritin (Safety Analysis Set)

**
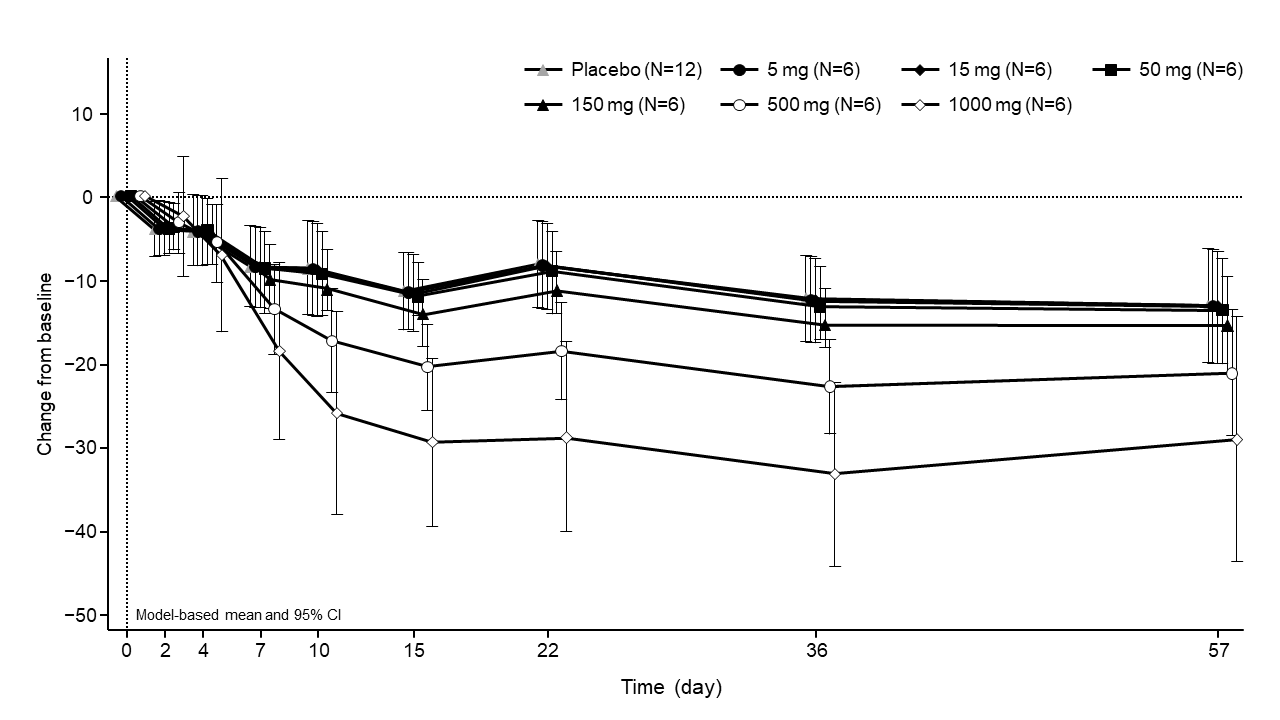
**

Model-based means and associated 95% CIs are derived from a mixed model for repeated measures for change from baseline. The model includes baseline value (continuous), dose (continuous), time (categorical), and the interaction between dose and time as fixed effects. An unstructured covariance structure is assumed for the within-subject covariance.
CI, confidential interval.

**Supplemental Figure S2.** Time course of change from baseline in serum iron (Safety Analysis Set)


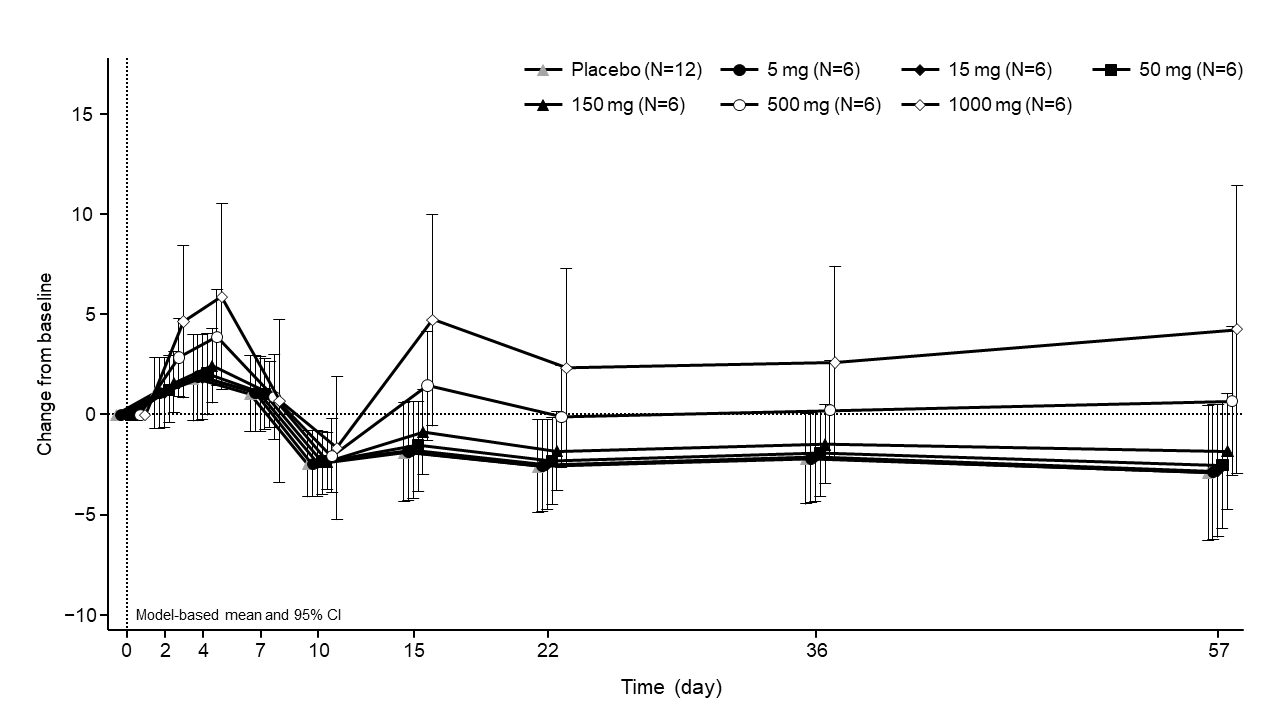


Model-based means and associated 95% CIs are derived from a mixed model for repeated measures for change from baseline. The model includes baseline value (continuous), dose (continuous), time (categorical), and the interaction between dose and time as fixed effects. An unstructured covariance structure is assumed for the within-subject covariance.
CI, confidential interval.

**
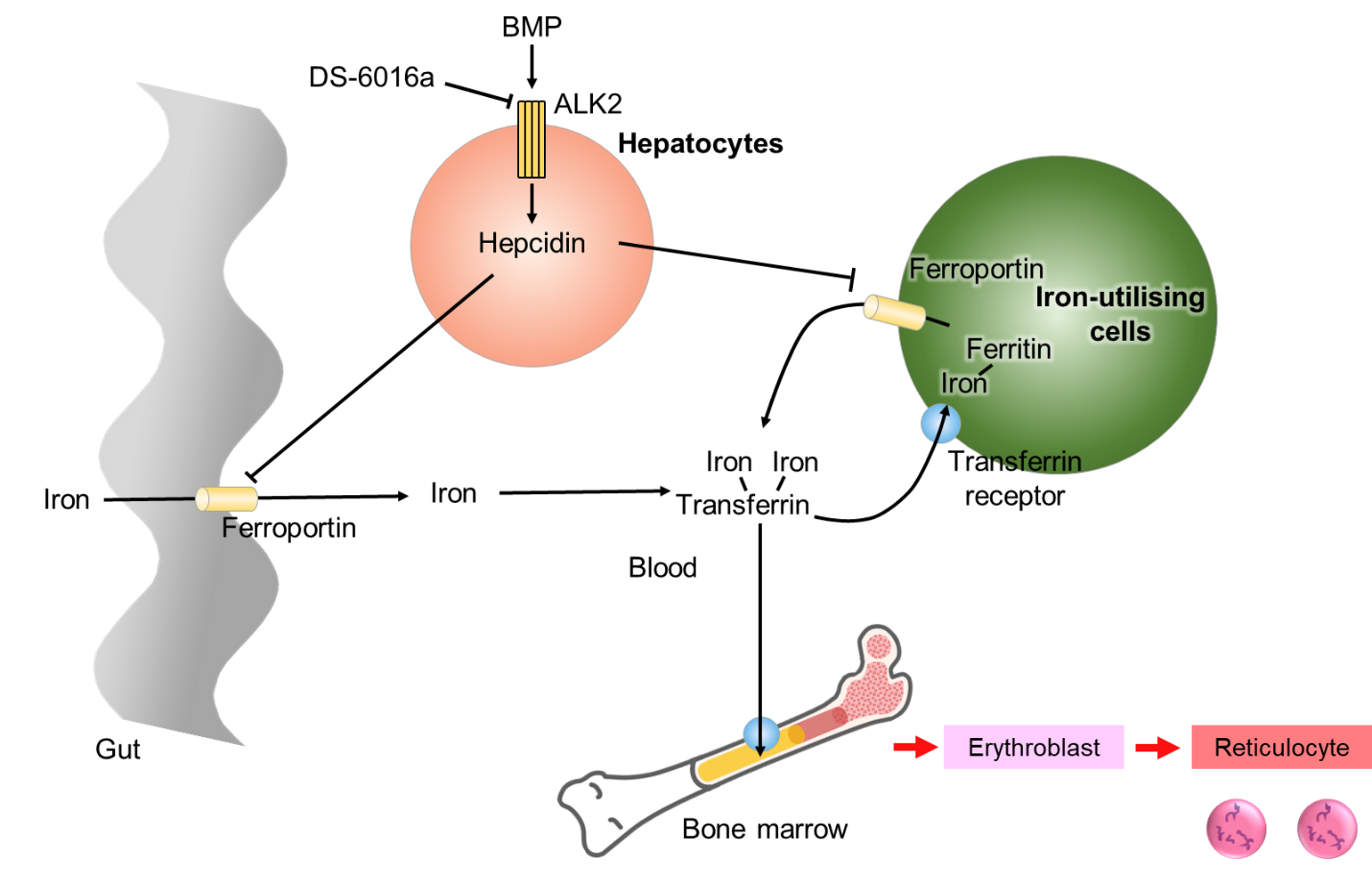
Supplemental Figure S3.** Relationship between iron, ferritin, hepcidin, ferroportin, and reticulocytes.

ALK2, activin receptor-like kinase 2; BMP, bone morphogenetic protein
